# Th2 Cytokines Increase the Expression of Fibroblast Growth Factor 21 in the Liver

**DOI:** 10.3390/cells10061298

**Published:** 2021-05-24

**Authors:** Seul-Gi Kang, Seong-Eun Lee, Min-Jeong Choi, Joon-Young Chang, Jung-Tae Kim, Ben-Yuan Zhang, Yea-Eun Kang, Ju-Hee Lee, Hyon-Seung Yi, Minho Shong

**Affiliations:** 1 Research Center for Endocrine and Metabolic Diseases, School of Medicine, Chungnam National University, 282 Munhwaro, Daejeon 35015, Korea; seulgikang@cnu.ac.kr (S.-G.K.); seongeun316@cnu.ac.kr (S.-E.L.); minjeongchoi@cnu.ac.kr (M.-J.C.); mitoupr77@cnu.ac.kr (J.-Y.C.); wjdxo3703@cnu.ac.kr (J.-T.K.); zhangbenyuan2019@gmail.com (B.-Y.Z.); yeeuni220@cnuh.co.kr (Y.-E.K.); serenaj@cnu.ac.kr (J.-H.L.); 2 Department of Medical Science, School of Medicine, Chungnam National University, 266 Munhwaro, Daejeon 35015, Korea; 3 Translational Immunology Institute, Chungnam National University, 266 Munhwaro, Daejeon 35015, Korea

**Keywords:** interleukin-4, interleukin-13, STAT6, FGF21, Th2 cytokines, hepatokine

## Abstract

Interleukin-4 (IL-4) and IL-13 are the major T helper 2 (Th2) cytokines, and they are involved in the regulation of metabolism in the adipose tissue. The liver contains diverse innate and adaptive immune cells, but it remains to be determined whether Th2 cytokines modulate energy metabolism in the liver. Here, using gene expression data from the Gene Expression Omnibus (GEO) and the BXD mouse reference population, we determined that the Th2 cytokines IL-4 and IL-13 increase the secretion of fibroblast growth factor 21 (FGF21) in the liver. In vitro experiments confirmed that FGF21 was highly expressed in response to IL-4 and IL-13, and this response was abolished by the Janus kinase (JAK)-signal transducer and activator of transcription 6 (STAT6) blockade. Moreover, FGF21 expression in response to Th2 cytokines was augmented by selective peroxisome proliferator-activated receptor α (PPARα) inhibition. In vivo administration of IL-4 increased FGF21 protein levels in the liver in a STAT6-dependent manner, but FGF21 secretion in response to IL-4 was not observed in the epididymal white adipose tissue (eWAT) despite the activation of STAT6. Intraperitoneal administration of IL-33, an activator of type 2 immune responses, significantly increased the level of FGF21 in the serum and liver after 24 h, but repeated administration of IL-33 attenuated this effect. Taken together, these data demonstrate that the IL-4/IL-13–STAT6 axis regulates metabolic homeostasis through the induction of FGF21 in the liver.

## 1. Introduction

Type 2 immune responses involve the secretion of type 2 cytokines, including interleukin (IL)-4, IL-5, IL-9, and IL-13, by T helper 2 (Th2) cells, which include eosinophils, mast cells, basophils, type 2 innate lymphoid cells (ILC2), and M2 macrophages [1,2]. Type 2 immunity is generally considered to have a regulatory function, to limit the injurious consequences of type 1-mediated immunity [1]. Previously, obesity was shown to promote a type 1 inflammatory response, including infiltration of activated T cells, expansion of M1 macrophages, and secretion of inflammatory cytokines, including tumor necrosis factor-α (TNF-α), IL-6, and IL-1β [3,4,5]. However, very little data are available on the involvement of Th1 immune responses in fatty liver disease. Recent studies have identified the pivotal role of type 2 immunity, including regulatory T cells (Treg), Th2 cells, and ILC2, in metabolic disease. These cells suppress inflammatory responses through production of the anti-inflammatory cytokine IL-10, which contributes to the improvement of insulin resistance [6,7]. There is emerging evidence that the IL-33-driven ILC2–eosinophil axis plays a role in metabolic homeostasis via IL-4/IL-13-mediated M2-like polarization of macrophages [8,9], thereby preventing the development of pro-inflammatory responses in metabolic disease. Although there has been pioneering work on the regulatory role of Th2 and ILC2 cells, how ILC2- and Th2-derived cytokines affect systemic glucose metabolism is not well understood.

FGF21 is the primary endogenous agonist of the FGF21 receptor, which is composed of the co-receptors FGF receptor 1 and β-Klotho [10,11]. FGF21 is highly expressed in the mouse adult liver [12] and was recently demonstrated to have beneficial metabolic effects such as promoting weight loss and improving glycemia [13]. The biology of FGF21 is intrinsically complicated due to its diverse metabolic functions in multiple target organs and its ability to act as an autocrine, paracrine, and endocrine factor. In the liver, FGF21 plays an important role in the regulation of fatty acid oxidation both in the fasted state and in mice consuming a high-fat, low-carbohydrate ketogenic diet [14,15]. FGF21 also regulates fatty acid metabolism in mice consuming a methionine- and choline-deficient diet that promotes hepatic lipotoxicity [16]. In white adipose tissue (WAT), FGF21 regulates glucose metabolism, and in susceptible WAT deposits, it can cause browning [17]. Peroxisome proliferator-activated receptor alpha (PPARα) agonists, including fenofibrate, Wy-14643, and GW7647, substantially increase hepatic FGF21 expression [18,19]. PPARα is also involved in fasting-associated hepatic glucose metabolism, as mice lacking PPARα showed no increase in FGF21 expression after fasting [14,20]. Nevertheless, FGF21 was still induced in these mice after consumption of a ketogenic diet, suggesting that other transcription factors might also regulate hepatic FGF21.

Among the Th2 cytokines, IL-4 and IL-13 are the major cytokines implicated in adipose tissue immune homeostasis and systemic energy metabolism [1]. IL-4 and IL-13 bind to the IL-4 receptor complex, which consists of the IL-4Rα chain and the common gamma chain (γc), leading to the phosphorylation of signal transducer and activator of transcription 6 (STAT6) by the receptor-associated kinases Janus-associated kinase (JAK)3 and Tyk2 or JAK2 [21,22]. Phosphorylation of STAT6 results in its dimerization and translocation to the nucleus, where it binds DNA promoter elements to regulate gene transcription. IL-4Ra is expressed by undifferentiated and differentiated adipocytes [23,24], and is involved in the differentiation of adipocytes into beige fat. Furthermore, it has been demonstrated that the IL-4–STAT6 immune axis controls peripheral nutrient metabolism and insulin sensitivity [25]. In this previous study, disruption of STAT6 decreased insulin activity, and activation of STAT6 by IL-4 improved insulin activity by attenuating adipose tissue inflammation. Although these findings identify a molecular link between the immune system and macronutrient metabolism, how Th2 cytokines control the glucose metabolism and insulin sensitivity remains unknown.

The liver plays a central role in the regulation of systemic glucose and lipid metabolism, which is maintained not only by metabolic pathways, including gluconeogenesis, glycogenolysis, lipogenesis, and fatty acid oxidation, but also by liver-derived secreted factors known as hepatokines [26,27]. To address the role of Th2 cytokines in systemic energy metabolism, we aimed to identify the hepatokines regulated in response to IL-4 and IL-13. We determined that FGF21 is secreted by hepatocytes in a STAT6-dependent manner in response to pharmacological IL-4 and IL-13 treatment or ILC2 activation. These studies demonstrate that Th2 cytokines IL-4 and IL-13 play an unexpected role in the regulation of FGF21 expression in the liver, suggesting a possible association between Th2 cytokines and metabolic homeostasis mediated by FGF21.

## 2. Materials and Methods

### 2.1. Animal Experiments

STAT6-deficient mice were purchased from Jackson Laboratory (Bar Harbor, ME, USA) and backcrossed onto a C57BL/6 background for more than 10 generations. Male mice at 8–9 weeks of age were used in all in vivo experiments. All mice were housed in a specific pathogen-free facility at the Chungnam National University Hospital Preclinical Research Center under controlled environmental conditions (a 12 h light/12 h dark cycle, an ambient temperature of 22 ± 2 °C, and 40–60% humidity) and fed a chow diet (Teklad Global 18% protein, 2918C, Envigo, Indianapolis, IN, USA). Mice were administered vehicle or recombinant mouse IL-4 (214-14, PeproTech, Rocky Hill, NJ, USA) via the intravenous (1 μg/mouse) or intraperitoneal (2 μg/mouse) route. Recombinant mouse IL-33 (0.5 μg/mouse, 080506, BioLegend, San Diego, CA, USA) was intraperitoneally administered to wild-type mice as previously described [28]. All experimental procedures complied with the institutional guidelines of the Animal Care and Use Committee and were approved by the Institutional Review Board of Chungnam National University Hospital (CNUH-019-P0087, Daejeon, Korea).

### 2.2. Isolation of Primary Hepatocytes

Primary hepatocytes were isolated from 8-week-old male mice as previously described [29]. Briefly, mice were perfused with EGTA solution (0.5 mM EGTA, 25 mM Tricine, 5.4 mM KCl, 0.44 mM KH2PO4, 140 mM NaCl, and 0.34 mM Na2HPO4; pH 7.2), followed by collagenase solution (0.8 mg/mL collagenase type I (Worthington, Freehold, NJ, USA) in Hank’s Balanced Salt Solution). The perfused livers were dissociated and filtered through 70 μm cell strainers (BD Falcon, Millville, NJ, USA) and then centrifuged at 1000× *g* for 5 min. The cells were gently resuspended and mixed with 40% Percoll solution (GE Healthcare, Buckingham, UK). After centrifugation at 12,000× *g* for 10 min at 4 °C, the isolated primary hepatocytes were cultured in Medium 199 (M4530, Sigma-Aldrich, St. Louis, MO, USA) with 10% fetal bovine serum (Thermo Fisher Scientific, Waltham, MA, USA) and 1% penicillin/streptomycin (LS202-02, Welgene, South Korea). Trypan blue exclusion showed that the cells seeded into 6-well plates (2*106/well) were more than 90% viable. Recombinant mouse IL-4 (100 ng/mL; 404-ML, R&D Systems, Minneapolis, MN, USA) and recombinant mouse IL-13 (100 ng/mL; 413-ML, R&D Systems) were used for the in vitro experiments. Cells were pretreated with JAK inhibitor I (1 μM; 420099, Merck Millipore, Burlington, MA, USA) 30 min before the addition of recombinant IL-4 or IL-13 or pretreated with fenofibrate (5 μM; F6020, Sigma Aldrich) and GW6471 (5 μM; G5045, Sigma Aldrich) 2 h before the addition of vehicle or Th2 cytokines.

### 2.3. Western Blot Analysis

Primary hepatocytes and mouse tissues were homogenized in cold lysis buffer (50 mM Tris-HCl, 150 mM NaCl, 1 mM EDTA, and 0.1% Triton X-100) with a protease inhibitor cocktail (#11836145001, Roche, Basel, Switzerland) and phosphatase inhibitors (#04906837001, Roche). Samples were centrifuged at 16,000× *g* for 15 min at 4 °C and measured by bicinchoninic acid (BCA) protein assay (#23227, Thermo Fisher Scientific). A total of 30–50 μg of protein was used for electrophoresis and transferred to methanol-activated polyvinylidene difluoride (PVDF) membranes (#IPVH00010, Merck Millipore). After blocking with 5% skim milk in Tris-buffered saline with 0.1% Tween, the membranes were incubated with specific primary and secondary antibodies. The anti-β-actin antibody (A2066) was from Sigma-Aldrich. Anti-phospho-STAT6 (#9361), anti-STAT6 (#9362), anti-phospho-STAT3 (#9131), and anti-STAT3 (#9139) antibodies were purchased from Cell Signaling Technology (Danvers, MA, USA). The anti-FGF21 antibody (NBP1-59291) was obtained from Novus Biologicals (Littleton, CO, USA). Anti-mouse IgG (#7076) and anti-rabbit IgG (#1706515) secondary antibodies were purchased from Cell Signaling Technology and Bio-Rad (Hercules, CA, USA), respectively. All primary antibodies were diluted 1:1000 in a total volume of 10 mL (5% skim milk in Tris-buffered saline with 0.1% Tween) and secondary antibodies were diluted 1:5000 in a total volume of 10 mL (5% skim milk in Tris-buffered saline with 0.1% Tween). The immunoreactive images were obtained using an ODYSSEY imaging system (LI-COR biosciences, Lincoln, NE, USA). To obtain the relative band density, the signal intensity of target proteins was quantified through Image Studio Software (v4.0, LI-COR biosciences). The band density of target proteins was normalized to those of β-actin.

### 2.4. Quantitative PCR Analysis

TRIzol reagent (15596018, Thermo Fisher Scientific) was used for the extraction of total RNA from primary hepatocytes or mouse tissues. cDNA was synthesized from 5 μg of total RNA using oligo(dT)_15_ primers (C1101, Promega, Madison, WI, USA) and M-MLV reverse transcriptase (28025, Thermo Fisher Scientific). Quantitative PCR analysis was performed with the synthesized cDNA, SYBR Green PCR Master Mix (Applied Biosystems, Foster City, CA, USA), a 7500 Fast Real-Time PCR System (Applied Biosystems), and the following primers: *Fgf21* forward, 5′-AGATCAGGGAGGATGGAACA-3′; *Fgf21* reverse, 5′-TCAAAGTGAGGCGATCCATA-3′; *18s* forward, 5′-CTGGTTGATCCTGCCAGTAG-3′; and *18s* reverse, 5′-CGACCAAAGGAACCATAACT -3′. Relative quantification was calculated according to the ∆∆CT method using Applied Biosystems 7500 Software (ver. 2.0.6) with normalization to *18s* rRNA and expressed as the fold change relative to the vehicle control.

### 2.5. Measurement of the FGF21 Protein Concentration

Cell supernatant was obtained from primary hepatocytes by centrifugation at 16,000× *g* for 5 min. Blood was collected from the retro-orbital sinus and allowed to clot for 2 h at room temperature. Samples were centrifuged at 600× *g* for 5 min and the serum was used for the measurement of FGF21 protein (MF2100, R&D Systems) according to the manufacturer’s instructions.

### 2.6. Gene Expression Analysis

The following gene sets in the Gene Expression Omnibus (GEO) were used to identify gene sets related to hepatic Th2 cytokines: GSE70705 [30] (hepatic gene expression in wild-type and *Il4rα*-deficient mice overexpressing IL-13, obtained using expression array profiling) and GSE95428 [31] (gene expression in the livers of wild-type and *Il4*-deficient mice, obtained using high throughput sequencing expression profiling). For GSE70705, sample signals were processed by GenomeStudio software (v.2011.1, Illumina Inc., San Diego, CA, USA) and the values were normalized to 500 based on the median and then converted to a log_2_ scale using the NIH mAdb microArray database). Statistical analysis was performed using the TM4 Mev Suite. For GSE95428, samples were converted to reads per million (RPM), shifted by +1, and then converted to a log_2_ scale. Based on these data, a gene set associated with STAT6 activation in the liver was identified. This signature included genes involved in proliferation (*Egr1*, *Junb*, and *H19*), lipid metabolism (*Cidea*, *Cideb*, *Cidec*, *Plin2*, *Acly*, and *Fasn*), fatty acid oxidatin (*Lipc*, *Cpt1a*, *Acads*, *Acadm*, *Acadl*, *Acadvl*, and *Hadh*), gluconeogenesis (*Pepck* and *G6pc*), and hepatokine secretion (*Gdf15* and *Fgf21*). Welch’s *t*-tests and ANOVA were used for statistical analysis.

### 2.7. Bioinformatics Analysis Using the BXD Mouse Genetic Reference Population

To analyze correlations in the expression of hepatic genes, we used the BXD recombinant inbred (RI) mouse strains, which are widely used as genetic reference populations. GeneNetwork (www.genenetwork.org), a database offering multi-omics data of BXD RI strains [32], was used to analyze correlations in the expression of *Il4*, *Il4rα*, *Fgf21*, and *Gdf15* in the livers of BXD mice fed a chow diet (GeneNetwork accession number, GN859). BXD RI mice were first divided into two groups according to the differential expression values of hepatic *Il4* or *Il4rα* (representing the top 25% group and the bottom 25% group; 10 mice/group), and the mean expression of hepatic *Fgf21* and *Gdf15* was compared in the lowest (bottom 25%) and the highest (top 25%) *Il4* or *Il4rα* expression groups, respectively. The data were analyzed by Student’s two-tailed *t*-tests.

### 2.8. Statistical Analysis

All results are presented as the mean ± standard errors of the mean (SEM). Statistical analyses were performed using IBM SPSS Statistics software (ver. 24, IBM, Armonk, NY, USA). Experimental data were analyzed using Student’s two-tailed *t*-tests when the values showed homogeneity of variance (Levene’s test) or one-way ANOVA followed by Tukey’s post hoc tests. A *p*-value of less than 0.05 was considered to represent statistical significance.

## 3. Results

### 3.1. Gene Expression Profiling in the Liver Reveals That Fgf21 Is Modulated by Th2 Cytokines

**Figure 1:**
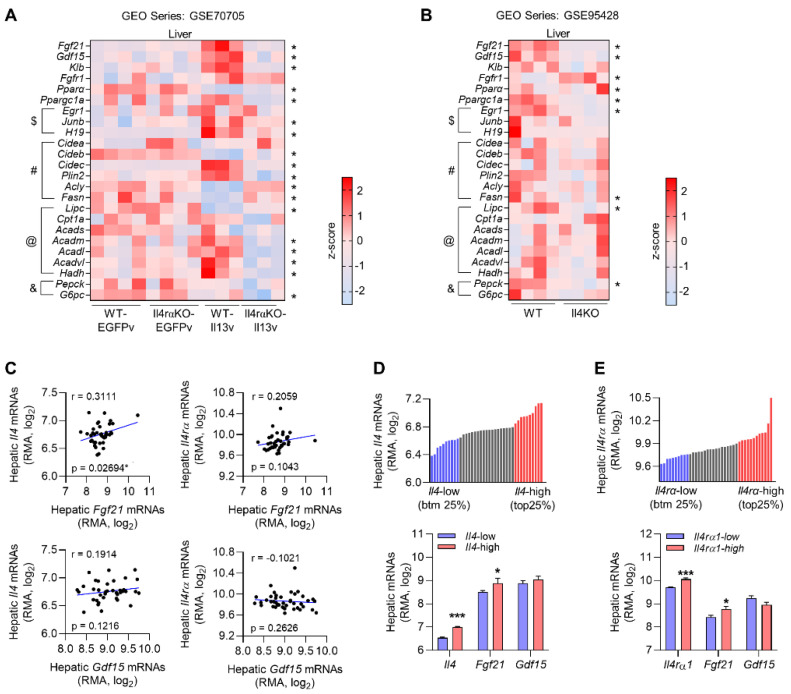
Gene expression profiling suggests that FGF21 is associated with Th2 cytokines. (**A**,**B**) Heat map showing the relative expression of hepatokines (*Fgf21*, *Gdf15*), FGF21 receptor components (*Klb*, *Fgfr1*), and genes associated with proliferation (indicated by the $ symbol), lipid metabolism (#), fatty acid oxidation (@), and gluconeogenesis (&) in the liver of *Il4rα*-deficient mice with or without IL-13 overexpression (GSE70705) (**A**) or in *Il4*-deficient mice (GSE95428) (**B**). (**C**) Correlation analysis of the relationship between the expression of hepatic *Il4*/*Il4rα* and hepatokines in BXD RI mice strains fed a chow diet (GN859, *n* = 39). (**D**) Hepatic *Il4* expression in 39 BXD strains (top panel). Mice were subdivided into the top 25% (*Il4*-high, *n* = 10) and the bottom 25% (*Il4*-low, *n* = 10) according to the *Il4* expression in the liver. The expression of hepatokines was compared in the two groups (bottom panel). (**E**) Hepatic *Il4rα* expression in 39 BXD strains (top panel). The expression of hepatokines was compared in mice divided into two groups, as described for (**D**) (bottom panel). Data in (**D**,**E**) are the mean ± SEM. Statistical analyses were performed using Welch’s *t*-tests and one-way ANOVA in (**A**,**B**) and Student’s *t*-tests in (**C**–**E**). * *p* < 0.05, *** *p* < 0.001 vs. control.

To identify the hepatokines induced in the response to Th2 cytokines, we analyzed previously published gene expression studies that evaluated the hepatic response to IL-13 overexpression [30] or genetic ablation of IL-4 in mice [31]. The hepatokines known to improve systemic energy metabolism include angiopoietin-like protein 6 (ANGPTL6) [33], adropin [34,35], FGF21 [14,36,37], growth differentiation factor 15 (GDF15) [38,39], and sex hormone-binding globulin (SHBG) [40,41], but only FGF21 and GDF15 were commonly enriched in these studies. Mice administered an IL-13 overexpression plasmid showed induction of *Fgf21* and *Gdf15* in the liver (Figure 1A), and conversely, the levels of these hepatokines were decreased in *Il4rα*-deficient mice. The co-receptor β-Klotho, a determinant of tissue specificity of FGF21 signaling whose expression is restricted in metabolic tissues [10,42], was significantly increased after overexpression of IL-13. Th2 cytokines activate the STAT6 signaling pathway to regulate hepatocyte proliferation [43] and anabolic lipid metabolism, including inhibition of fatty acid oxidation [25] and stimulation of lipogenesis [44] in the liver. Indeed, the expression of *Pparα*, a key regulator of fatty acid oxidation, was attenuated, and the expression of genes involved in proliferation (*Junb* and *H19*) and lipid metabolism (cell death-inducing DFFA-like effector c (*Cidec*), perilipin 2 (*Plin2),* and lipase C (*Lipc*)) was significantly upregulated, in the liver of IL-13-overexpressing mice. Consistent with this, another gene expression analysis of the liver suggested that hepatic expression of *Fgf21* and *Gdf15* was significantly decreased in *Il4*-deficient mice fed a chow diet compared with controls (Figure 1B).

To determine whether the expression of hepatokines correlates with *Il4* expression in various mouse populations, we analyzed gene expression data from the BXD RI mouse strains, which are widely used genetic reference populations [32]. Correlation analysis indicated that hepatic *Fgf21* expression was significantly associated with hepatic *Il4* expression (*r* = 0.3511) but not *Il4rα* expression. Moreover, hepatic *Gdf15* did not correlate with either hepatic *Il4* or *Il4rα* (Figure 1C). The mice were divided into two groups according to the hepatic *Il4* or *Il4rα* expression (the top 25% vs. the bottom 25%; *n* = 10 mice/group), and hepatic *Fgf21* expression was significantly increased in the group of mice with the highest *Il4* or *Il4rα* expression (Figure 1D,E). Taken together, these results suggest that expression of hepatic *Fgf21* is associated with Th2 cytokine expression in the liver.

### 3.2. IL-4 and IL-13 Increase FGF21 Expression in Primary Hepatocytes in a STAT6-Dependent Manner

**Figure 2:**
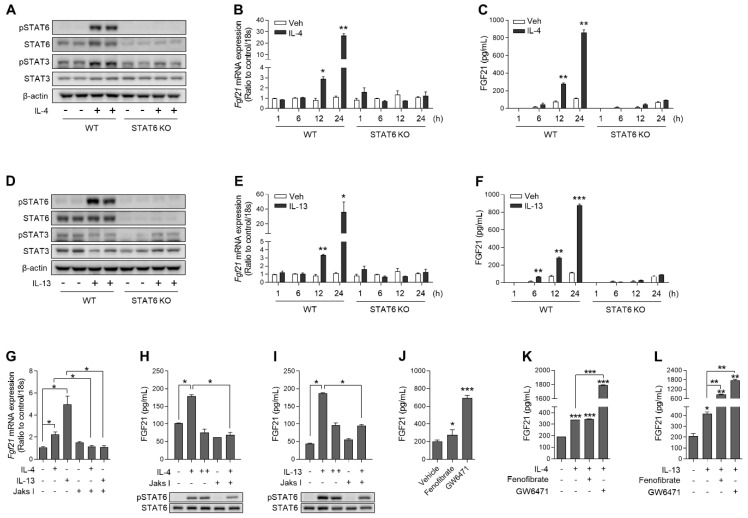
The IL-4/IL-13–JAK–STAT6 axis induces the expression of FGF21 in primary hepatocytes. (**A**) Western blot analysis showing IL-4-induced STAT signaling in wild-type or *Stat6*-deficient primary hepatocytes stimulated with recombinant mouse IL-4 (rIL-4) for 24 h. (**B**) Quantitative PCR analysis of *Fgf21* in primary hepatocytes after treatment with rIL-4 (*n* = 3). (**C**) The concentration of FGF21 in the culture medium of hepatocytes stimulated with rIL-4 (*n* = 3). (**D**) Western blot analysis showing IL-13-induced STAT signaling in wild-type or STAT6-deficient primary hepatocytes stimulated with recombinant mouse IL-13 (rIL-13) for 24 h. (**E**) Quantitative PCR analysis of *Fgf21* in primary hepatocytes after treatment with rIL-13 (*n* = 3). (**F**) The concentration of FGF21 in the culture medium of hepatocytes stimulated with rIL-13 (*n* = 3). (**G**) Quantitative PCR analysis of *Fgf21* in primary hepatocytes treated with rIL-4, rIL-13, and JAK inhibitor I (1 μM; 1 h pre-treatment) for 12 h (*n* = 3). (**H**,**I**) The concentration of FGF21 in the culture medium of hepatocytes co-treated with rIL-4 (+, 100 ng/mL; ++, 200 ng/mL) and JAK inhibitor I (**H**) or rIL-13 (+, 100 ng/mL; ++, 200 ng/mL) and JAK inhibitor I (**I**) for 24 h (*n* = 3). (**J**) The concentration of FGF21 in the culture medium of hepatocytes stimulated with fenofibrate (5 μM) or GW6471 (5 μm) for 24 h (*n* = 3). (**K**,**L**) The concentration of FGF21 in the culture medium of co-treated hepatocytes (*n* = 3). The concentration of rIL-4 and rIL-13 used in (**A**–**G**,**K**,**L**) is 100 ng/mL. Data are the mean ± SEM. Statistical analyses were performed using Student’s *t*-tests in (**B**,**C**,**E**,**F**,**J**) and one-way ANOVA followed by Tukey’s post hoc tests in (**G**–**I**,**K**,**L**). * *p* < 0.05, ** *p* < 0.01, *** *p* < 0.001 vs. control.

To verify whether FGF21 was induced through the canonical Th2 cytokine signaling pathway, primary hepatocytes were isolated from wild-type and global *Stat6*-deficient mice. Recombinant IL-4 (rIL-4) treatment induced STAT6 and STAT3 phosphorylation in wild-type primary hepatocytes, but this was attenuated in *Stat6*-deficient primary hepatocytes (Figure 2A). Treatment of cultured hepatocytes derived from wild-type mice with rIL-4 increased the expression of FGF21 mRNA and protein in a time-dependent manner, but the effects of rIL-4 were attenuated in primary *Stat6*-deficient hepatocytes (Figure 2B,C). Consistent with the effects of rIL-4, rIL-13 increased STAT6 phosphorylation only in wild-type hepatocytes (Figure 2D). Next, we examined the effects of rIL-13 on the expression of FGF21, and found that both FGF21 mRNA and protein were enhanced by rIL-13 in a STAT6-dependent manner (Figure 2E,F). Treatment of wild-type hepatocytes with JAK inhibitor I, a non-selective JAK inhibitor, inhibited the increased expression of *Fgf21* by rIL-4 or rIL-13 (100 ng/mL) (Figure 2G). Similarly, co-treatment with Th2 cytokines and Jak I attenuated STAT6 phosphorylation compared with the treatment of Th2 cytokines alone. FGF21 levels in the cellular supernatant were increased by treatment with rIL-4 or rIL-13 (100 ng/mL), but this response was not dose-dependent (Figure 2H,I). Consistently, JAK inhibition reduced the secretion of FGF21 in response to Th2 cytokines, suggesting that canonical IL-4/IL-13 signaling was involved in the induction of FGF21 in hepatocytes.

A previous study showed that IL-4-induced STAT6 physically associates with PPARα and represses its transcriptional activity [25]. As expected, both fenofibrate (a PPARα agonist) and GW6471 (a PPARα antagonist) enhanced FGF21 secretion (Figure 2J). Co-treatment of wild-type hepatocytes with rIL-4 and GW6471 indicated that inhibition of endogenous PPARα accelerated IL-4-mediated induction of FGF21 (Figure 2K). A similar effect was observed for rIL-13 treatment (Figure 2L). Taken together, these data demonstrate that the IL-4/IL-13–STAT6 axis can promote hepatic FGF21 production independently of PPARα, although hepatic FGF21 production is accelerated by the PPARα antagonist GW6471.

### 3.3. IL-4 Administration in Mice Increases FGF21 Production in the Liver, but Not the Epididymal White Adipose Tissue

**Figure 3:**
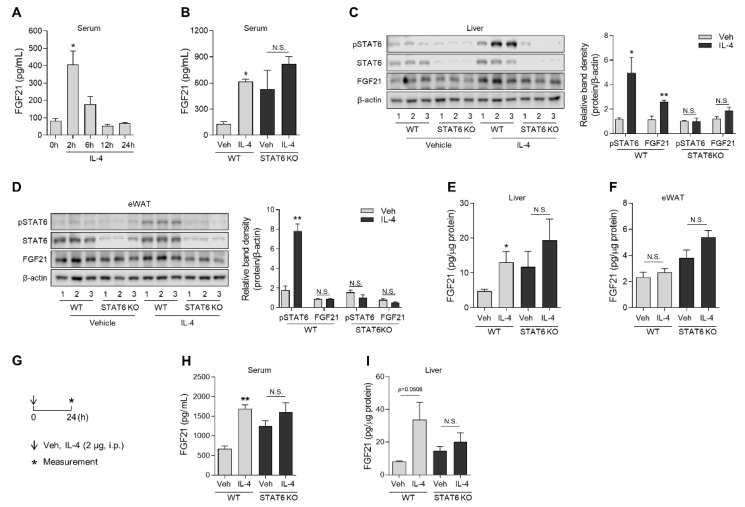
FGF21 production increased in the serum and liver in mice administered rIL-4. (**A**) The serum concentration of FGF21 in mice intravenously administered rIL-4 (1 μg/mouse, *n* = 3). (**B**) The serum concentration of FGF21 in wild-type or *Stat6*-deficient mice 2 h after rIL-4 administration (*n* = 3). (**C**) Western blot analysis of STAT6 and FGF21 in the liver of mice treated with vehicle or rIL-4 (left panel) and the relative band densities of phospho-STAT6 and FGF21 (right panel). (**D**) Western blot analysis of STAT6 and FGF21 in the eWAT of mice administered vehicle or rIL-4 (left) and the relative band densities of phospho-STAT6 and FGF21 (right). (**E**,**F**) The concentration of FGF21 protein in the liver (**E**) and eWAT (**F**) of wild-type or *Stat6*-deficient mice administered rIL-4 (*n* = 3). (**G**) Schematic overview of the intraperitoneal administration strategy in mice. (**H**) The serum concentration of FGF21 in wild-type or *Stat6*-deficient mice injected intraperitoneally with rIL-4 (2 μg/mouse, *n* = 3). (**I**) The concentration of FGF21 protein in the liver of mice administered rIL-4. Data are the mean ± SEM. Statistical analyses were performed by one-way ANOVA followed by Tukey’s post hoc tests in (**A**) and Student’s *t*-tests in (**B**–**F**,**H**,**I**). * *p* < 0.05, ** *p* < 0.01, *** *p* < 0.001 vs. control. N.S., not significant.

The in vitro experiments described above led us to investigate whether the increase in FGF21 in response to Th2 cytokines is also observed in vivo. To observe the acute response to Th2 cytokines, mice were intravenously administered rIL-4 (1 μg/mouse) via the tail vein, and serum levels of FGF21 were measured in a time-course. rIL-4 administration significantly increased serum FGF21 in wild-type mice after 2 h (Figure 3A). Next, rIL-4 was intravenously administered to wild-type and *Stat6*-deficient mice. As observed previously, rIL-4 clearly increased serum FGF21 in wild-type mice 2 h after injection (Figure 3B). Although *Stat6* deficiency enhanced the basal serum FGF21 concentration, as previously reported [25], the serum FGF21 level was unresponsive to rIL-4 stimulation in mice with STAT6 deficiency. STAT6 phosphorylation occurred after acute rIL-4 treatment in both the liver and the epididymal white adipose tissue (eWAT) of wild-type mice, but FGF21 was significantly increased only in the liver by western blot analysis (Figure 3C,D). Consistent results were observed by measuring the FGF21 protein concentration in the tissue (Figure 3E,F). As delivery efficiency into the tissue can depend on the administration route [45,46], mice were next intraperitoneally injected with rIL-4 and sacrificed 24 h after injection (Figure 3G). In this experiment as well, serum FGF21 was increased by rIL-4 in wild-type mice (Figure 3H), and the FGF21 protein concentration in the liver also tended to increase (Figure 3I). These data indicate that IL-4/STAT6-dependent induction of FGF21 is a specific phenomenon observed in the liver.

### 3.4. A Single Dose of IL-33 Increases FGF21 Protein Expression in Various Tissues in Mice

**Figure 4:**
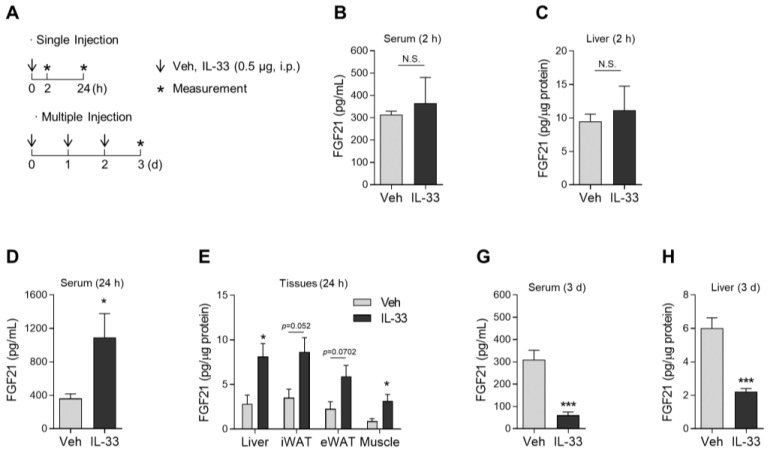
Administration of rIL-33 increases FGF21 in the serum and tissues. (**A**) Schematic overview of the intraperitoneal administration strategy in mice. (**B**,**C**) The concentration of FGF21 in the serum (**B**) and liver (**C**) of wild-type mice 2 h after intraperitoneal administration of rIL-33 (500 ng/mouse, *n* = 3). (**D**,**E**) The concentration of FGF21 protein in the serum (**D**) and liver (**E**) of mice 24 h after rIL-33 administration (*n* = 3). (**F**,**G**) The concentration of FGF21 in the serum (**F**) and liver (**G**) of mice administered rIL-33 daily for 3 days (*n* = 3). Data are the mean ± SEM. Statistical analyses were performed using Student’s *t*-tests. * *p* < 0.05, *** *p* < 0.001 vs. control. N.S., not significant.

IL-33, a member of the IL-1 superfamily, is mainly produced by epithelial and endothelial cells and can be either pro-inflammatory or anti-inflammatory depending on the tissue and disease context [47]. With respect to its anti-inflammatory effects, IL-33 activates type 2 immune cells, including ILC2 and eosinophils, resulting in the production of IL-4, IL-5, and IL-13 [48,49]. To stimulate the endogenous production of Th2 cytokines, wild-type mice were administered single or multiple intraperitoneal injections of rIL-33 (0.5 μg/mouse) (Figure 4A). First, we analyzed the levels of FGF21 in the serum and metabolic tissues 2 or 24 h after rIL-33 administration. Although the production of FGF21 protein was unaffected 2 h after rIL-33 treatment (Figure 4B,C), FGF21 levels were significantly increased in the serum, liver, and skeletal muscle 24 h after rIL-33 administration (Figure 4D,E). However, the effect of rIL-33-mediated FGF21 production was dramatically reversed in mice injected with rIL-33 daily for 3 days (Figure 4G,H), indicating that excessive administration of rIL-33 attenuates FGF21 production in mice. Taken together, these data indicate that a single injection of rIL-33 can enhance FGF21 protein levels in wild-type mice.

## 4. Discussion

In this study, we focused on the role of Th2 cytokines in STAT6-mediated FGF21 production. Our findings indicate that STAT6 signaling is a key mechanism in IL-4- or IL-13-induced hepatic FGF21 production in vitro and in vivo. This suggests that FGF21 plays an essential role in the metabolic improvements observed in response to treatment with Th2 cytokines. The regulatory function of Th2 cytokines is an important mechanism underlying systemic glucose homeostasis. IL-4 and IL-13, which are both induced by type 2 immune responses, decrease inflammation in metabolic organs by inducing M2 polarization of macrophages, leading to the improvement of systemic glucose intolerance in mice [50]. However, it remains to be determined how Th2 cytokines regulate the expression of hormetic factors, which in turn improve systemic glucose homeostasis. Here, we found that FGF21 is a critical hepatokine in Th2 cytokine-mediated immunoregulatory responses, which may ameliorate metabolic impairments caused by obesity and insulin resistance.

FGF21 expression is regulated by a variety of nutrient stresses, such as starvation, a ketogenic diet, amino acid deprivation, undernutrition or malnutrition, and a high-fat diet or obesity. PPARα is a well-established transcriptional activator of FGF21. It stimulates fasting-induced ketogenesis, lipolysis, and fatty acid oxidation in the liver [19], thereby contributing to the reduction of hepatic steatosis [14]. Co-transcriptional activators interacting with PPARα, cAMP-responsive element binding protein H (CREBH) [51], and carbohydrate responsive element binding protein (ChREBP) [52], were found to upregulate the FGF21 expression in response to metabolic status. In a previous study, STAT6 interacted with PPARα, suppressing its transcriptional activity [25]. Nevertheless, STAT6 activation in response to rIL-4 treatment enhanced systemic insulin sensitivity and glucose disposal in a STAT6-dependent manner [25,53]. In the current study, co-treatment of primary hepatocytes with rIL-4 and a PPARα antagonist increased the induction of FGF21 compared with the effects of rIL-4 alone. Similar results were obtained with rIL-13. These observations raise the possibility that the insulin sensitivity observed in mice treated with rIL-4 [25] could be modulated by FGF21. Moreover, these previous studies suggest that rIL-4 stimulation of hepatocytes shifted the metabolic flux from fatty acid oxidation to glucose oxidation [25], indicating that STAT6 acts as a repressor of PPARα, and its activity may be increased in glucose-enriched conditions. Indeed, rIL-4 treatment enhanced glucose uptake and glycolysis in splenic B cells and increased the cellular ATP production, and this was not observed in STAT6-deficient B cells [54]. STAT6 also modulates pyruvate kinase M2 (PKM2), an essential enzyme for glycolysis, in the liver [55], suggesting that Th2 cytokines/STAT6 signaling facilitate systemic glucose metabolism, and that FGF21 induced by STAT6 signaling may be a modulator of improved insulin sensitivity.

In our study, induction of FGF21 in response to rIL-4 was only observed in the liver, not in the epididymal adipose tissue, even though phosphorylation of STAT6 after rIL-4 treatment was observed in both tissues. A previous study also reported that liver and subcutaneous white adipose tissue show differences in FGF21 transcription in response to physiological stimuli, including fasting [56] and cold [57]. *Fgf21* mRNA expression was markedly increased in the liver after 6 h of fasting and was maintained over a 24-h fasting period [56]. However, *Fgf21* expression in subcutaneous white adipose tissue was not affected by fasting for 6 or 24 h, and reversely increased after refeeding, suggesting that *Fgf21* expression can be differentially expressed in a tissue-specific manner. Similar to this study, acute cold exposure significantly increased hepatic *Fgf21* expression, although the increase was modest in epididymal white adipose tissue [57]. Moreover, the regulator involved in *Fgf21* induction might be modulated differently in different tissues. Indeed, Sp1, a mammalian transcriptional factor, binds to the *Fgf21* promoter and increases the expression of *Fgf21* [58]. Interestingly, Sp1 protein expression in mice with diet-induced obesity was much higher in white adipose tissue than in the liver. These differences in FGF21 expression between the liver and adipose tissue in response to a stimulus suggest that the transcriptional regulatory elements governing FGF21 expression differ between the liver and adipose tissue. In our study, rIL-4 administered intravenously significantly increased the expression of FGF21 in the serum and liver at 2 h post-injection, indicating that serum FGF21 is induced by rIL-4 derived from liver tissue. Consistent with this, treatment with rIL-33, which is known to induce the production of Th2 cytokines by ILC2 and eosinophils, significantly increased the production of FGF21 in the liver and skeletal muscle.

The STAT6 pathway is critical for maintaining cellular and systemic energy metabolism, particularly oxidative mitochondrial metabolism. Given that STAT6 localizes to the mitochondria [59], we hypothesized that activation of the STAT6 pathway may regulate the production of FGF21, a major mitokine. In this study, we found that STAT6 signaling is involved in the Th2 cytokine-mediated FGF21 expression in the liver. Treatment with recombinant IL-4 and IL-13 showed additive or synergistic effects on serum FGF21 levels in mice. This finding indicates that the STAT6 pathway is important for the induction of FGF21 expression in the liver, which is accelerated by PPARα antagonist treatment. However, further research is required to establish a functional role for STAT6-dependent FGF21 production with respect to that of PPARα-mediated induction using PPARα knockout animals.

Previous studies identified a Th1/Th2 cytokine imbalance in the metabolic tissues of mice with obesity and insulin resistance [60]. Interferon (IFN)-γ-producing Th1 cells are increased in the adipose tissue of mice with diet-induced obesity and obese human subjects [61,62]. By contrast, Th2 cells are predominantly increased in the metabolic organs of insulin-sensitive subjects [63,64]. Therefore, it has been suggested that strategies to restore the Th1/Th2 cytokine balance in metabolic tissues might be useful for treating diabetes and metabolic diseases. Our results also support this speculation by showing FGF21 production in response to treatment with Th2 cytokines in vitro and in vivo. Additionally, we showed in a previous study that GDF15, another mitokine, can be induced by Th2 cytokines/STAT6 signaling [24], thereby preventing metabolic impairment caused by obesity and insulin resistance.

## Data Availability

The data presented in this study are available on request from the corresponding author.
